# Association of Aortic Arch Calcification on Chest X-ray with Procedural Thromboembolism after Mechanical Thrombectomy for Acute Ischemic Stroke

**DOI:** 10.3390/medicina57090859

**Published:** 2021-08-24

**Authors:** Hoon Gi Kim, Sang Hyuk Lee, Taek Min Nam, Ji Hwan Jang, Young Zoon Kim, Kyu Hong Kim, Do-Hyung Kim, Seung Hwan Kim

**Affiliations:** 1 Department of Neurosurgery, Samsung Changwon Hospital, Sungkyunkwan University School of Medicine, Changwon 51353, Korea; commisarang@hanmail.net (H.G.K.); shlee858@naver.com (S.H.L.); taekmin82@gmail.com (T.M.N.); gebassist@naver.com (J.H.J.); yzkim@skku.edu (Y.Z.K.); Unikkh@unitel.co.kr (K.H.K.); 2 Department of Neurology, Samsung Changwon Hospital, Sungkyunkwan University School of Medicine, Changwon 51353, Korea; pons1217@gmail.com

**Keywords:** aortic arch, calcification, thromboembolism, mechanical thrombectomy, acute ischemic stroke

## Abstract

*Background and Objective*: Procedural thromboembolism after a mechanical thrombectomy (MT) for an acute ischemic stroke (AIS) has rarely been studied. It may occur from the artery-to-artery embolization of atherosclerotic plaque in the aortic arch. We investigated the relationship between aortic arch calcification (AoAC) on a chest X-ray and procedural thromboembolism on diffusion-weighted imaging (DWI) after an MT. *Materials and Methods*: From January 2017 to December 2020, 131 patients underwent DWI within two days following an MT for an AIS. Procedural thromboembolism was defined as new DWI-positive lesions in other territories from the occluded artery on DWI within two days after MT. *Results*: Procedural thromboembolism was observed in 30 (22.9%) patients. Procedural thromboembolism was associated with old age (72.3 ± 9.44 vs. 65.7 ± 12.8 years, *p* = 0.003), a longer procedural time (77.6 ± 37.6 vs. 60.1 ± 29.7 min, *p* = 0.024), and AoAC (calcification (73.3%) vs. no calcification (29.7%), *p* < 0.001). Multivariable logistic regression analysis showed that procedural thromboembolism was independently associated with AoAC (adjusted odds ratio (OR): 6.107, adjusted 95% confidence interval (CI): 2.374–15.705, *p* < 0.001) and a longer procedural time (adjusted OR: 1.015, adjusted 95% CI: 1.001–1.030, *p* = 0.031). *Conclusions*: Procedural thromboembolism after an MT for an AIS was related to AoAC on a chest X-ray and a longer procedural time. Our results suggest that although rapid recanalization is the most crucial goal of an MT for an AIS, the importance of the careful advance of the guiding catheter through the aortic arch should not be underestimated to reduce the risk of procedural thromboembolism, especially in patients with AoAC on a chest X-ray.

## 1. Introduction

A mechanical thrombectomy (MT) is a standard treatment for an acute ischemic stroke caused by large artery occlusion, due to its site specificity and high recanalization rates [1,2,3]. The endovascular approach has the advantage of faster and safer access to the cerebral arteries; however, it also has a risk of procedural thromboembolism [4,5,6]. It is well known that diffusion-weighted imaging (DWI) shows multiple small asymptomatic lesions after endovascular treatment of cerebrovascular disease [1,4,5,6,7]. Most previous studies on procedural thromboembolism in neurointervention have focused on coil embolization and carotid artery stenting [4,7,8]. As the goal of an MT for an acute ischemic stroke (AIS) is to achieve rapid recanalization, procedural thromboembolism after an MT for an AIC is considered not as crucial as recanalization [1]. Therefore, procedural thromboembolism after an MT for an AIS has rarely been studied.

In the field of neurointervention, including an MT for an AIS, procedural thromboembolism can be caused by atherosclerotic embolization from the aortic arch and adjacent large arteries, as the guiding catheters advance through the vessels [9,10,11,12]. Atherosclerosis of the aortic arch can be a source of procedural thromboembolism [5,12,13], and it can be assessed using a chest X-ray before the procedure [14]. We hypothesized that the aortic arch calcification (AoAC) on a chest X-ray could be related to procedural thromboembolism after an MT for an AIS.

This study investigated the relationship between AoAC on a chest X-ray and procedural thromboembolism after an MT for an AIS on DWI within two days after the MT.

## 2. Materials and Methods

### 2.1. Data Analysis

We conducted a retrospective analysis of patients who underwent MT for AIS in our hospital from January 2017 to December 2020. A total of 212 patients who met the inclusion criteria were included in this study. The inclusion criteria were as follows: (1) AIS with symptoms; (2) large artery occlusions confirmed using magnetic resonance (MR) angiography or computed tomography angiography; (3) ≤24 h from symptom onset to treatment; (4) at least one-half mismatch between cerebral blood flow and cerebral blood volume map with MR perfusion imaging; (5) treated by MT. Of the 212 patients, 81 patients were excluded due to the lack of DWI within two days after MT. Therefore, 131 consecutive patients were included.

The data on the baseline characteristics of the patients, treatment details, and clinical and radiological outcomes were obtained from medical records. The medical records and imaging data were reviewed after approval from the institutional review board (SCMC 2021-07-008). The baseline characteristics of the patients included age; sex; past medical history, including hypertension, diabetes mellitus, and atrial fibrillation; smoking status; AoAC on chest X-ray; aortic arch type; occlusion site; stroke etiology; occlusion site; tandem occlusion; National Institutes of Health Stroke Scale (NIHSS) score on admission; the time from symptom onset to door; the time from door to puncture; and the use of intravenous thrombolysis. Procedural details included each interventionalist; experience of the interventionalist; timing of MT, such as daytime, or nighttime or weekends; the time when the MT was performed, approach side; use of balloon guided catheter; use of intermediate catheter; whether rescue treatments, such as angioplasty or stenting, were performed; the total number of MT attempts; procedural time; and the incidence of procedural thromboembolism. The clinical and radiological outcomes included a 3-month modified Rankin Scale (mRS) score and thrombolysis in cerebral infarction (TICI) grades.

AoAC on chest X-ray was evaluated by two observers (HG Kim, and SH Kim) based on the presence of calcification. Both observers were neurosurgeons, not radiologists, and had insufficient experience in analyzing aortic arch calcification on chest X-ray; therefore, aortic arch calcification was assessed in a binary way, such as presence or absence. Aortic arch type was classified based on the vertical distance from the innominate artery to the top of the arch (Type 1 arch: distance of <1 diameter of the left common carotid artery (CCA); Type 2 arch: between 1 and 2 CCA diameters; and Type 3 arch: >2 CCA diameters) [10]. Stroke etiology was classified according to the trial of ORG 10172 in acute stroke treatment (TOAST) criteria. Each patient’s NIHSS score was assessed on admission. Patients admitted within 4.5 h of AIS symptom onset were considered suitable candidates for the intravenous tissue-plasminogen activator. Procedural time was defined as the total time from groin puncture to recanalization. Procedural thromboembolisms were defined as new DWI-positive lesions in other territories from the occluded artery on DWI within two days after MT. A good clinical outcome was defined as a 3-month mRS score of ≤2. Successful recanalization was defined as a TICI grade of 2b or 3.

### 2.2. MT Procedures

MT was conducted under conscious sedation through the femoral artery. The primary MT modality was decided on the basis of the surgeon’s discretion and conducted using catheter aspiration or a stent retriever, or a combination of the two methods. The use of a balloon-guided catheter (Flowgate, Stryker Neurovascular, Fremont, CA, USA) and intermediate catheter (Catalyst 6, Stryker Neurovascular, Mountain View, CA, USA) was decided based on the condition of the vessels. Rescue treatments, including balloon angioplasty and stenting, were considered when residual stenosis in atherosclerotic occlusion was observed on an angiograph performed after MT.

### 2.3. Statistical Analysis

The baseline characteristics, procedural details, and clinical and radiological outcomes were compared between patients with AoAC and those without AoAC, and between patients with procedural thromboembolism and those without procedural thromboembolism. Categorical variables were analyzed using the chi-square test or Fisher’s exact test. Continuous variables were analyzed using the Student’s *t*-test or the Mann–Whitney U test. Statistical significance was set at *p* < 0.05. Multivariable logistic regression analysis was used to evaluate factors affecting procedural thromboembolism after MT for AIS. Variables with *p* < 0.20 in the univariate analysis were included in the logistic regression analysis. All statistical analyses were conducted using SPSS version 22 (IBM Corp., Armonk, NY, USA).

## 3. Results

During the study period, 131 patients (79 men and 52 women; mean age 67.2 years; age range, 30–87 years) who underwent an MT for an AIS at our hospital were included in this study.

In our hospital, three neurosurgeons performed the neurointerventional procedures. The experience of each neurosurgeon as of 2020 is as follows: interventionalist one (5 years), interventionalist two (3 years), and interventionalist three (1 year). They have performed about 20–30 cases of MT for an AIS each year.

The aortic arch types were as follows: type one (*n* = 38), type two (*n* = 68), and type three (*n* = 25). The occlusion sites were the cervical internal carotid artery (ICA) in five patients, the cervical ICA and middle cerebral artery (MCA) in 12 patients, the ICA distal to cervical segment in 17 patients, the ICA distal to cervical segment and MCA in four patients, the M1 segment in 57 patients, the M1 and M2 segment in eight patients, the M2 segment in 16 patients, the anterior cerebral artery in one patient, the vertebral artery in three patients, and the basilar artery in eight patients.

**Figure 1:**
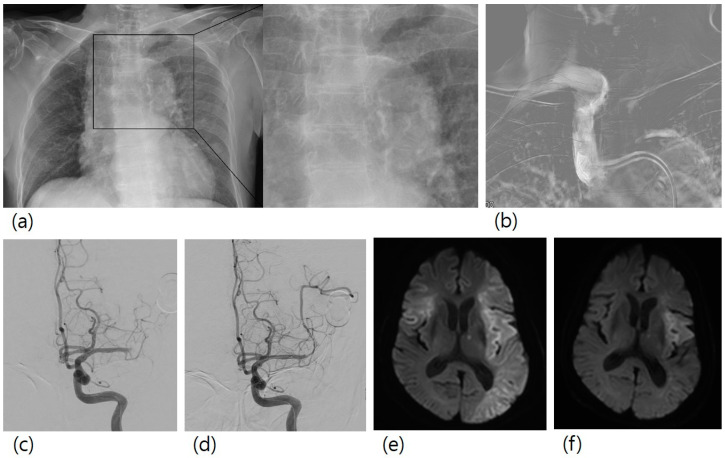
A case of mechanical thrombectomy for left M1 occlusion. A 78-year-old female patient with right hemiparesis visited our hospital. (**a**) Her chest X-ray showed aortic arch calcification. (**b**) In the first step of the procedure, guiding catheter was advanced to the brachiocephalic artery, and a roadmap image was obtained. (**c**) Left M1 occlusion was observed. (**d**) Mechanical thrombectomy with stent retriever was conducted, and successful recanalization was achieved. (**e**) Diffusion-weighted imaging two days after mechanical thrombectomy showed a new lesion in the right MCA territory, (**f**) which was not observed in diffusion-weighted imaging before mechanical thrombectomy.

Stroke etiology was atherosclerotic occlusions (*n* = 38), cardioembolic occlusions (*n* = 49), others (*n* = 2), and unknown (*n* = 42). The overall rate of AoAC was 39.7% (*n* = 52). Procedural thromboembolism occurred in 30 (22.9%) patients. All the patients with procedural thromboembolism showed no newly developed neurological symptoms. One patient had a large-sized procedural thromboembolism, and the other 29 patients had multiple focal lesions. Figure 1 shows a case of a patient with a large-sized procedural thromboembolism.

The baseline characteristics, procedural details, and clinical and radiological outcomes of the patients with AoAC and those without AoAC are summarized in Table 1. The patients with AoAC were significantly older than those without AoAC (71.3 ± 8.01 vs. 64.5 ± 14.0, *p* = 0.002). Atrial fibrillation was observed more frequently in the patients with AoAC than in those without AoAC (24/52 (46.2%) vs. 22/79 (27.8%), *p* = 0.040). Aortic arch type 2 or type 3 were observed more frequently in the patients with AoAC than in those without AoAC (46/52 (88.5%) vs. 47/79 (59.5%), *p* < 0.001). Procedural thromboembolism occurred more frequently in the patients with AoAC than in those without AoAC (22/52 (42.3%) vs. 8/79 (10.1%), *p* < 0.001).

Table 2 compares the baseline characteristics, procedural details, and clinical and radiological outcomes between the patients with procedural thromboembolism and those without procedural thromboembolism. The patients with procedural thromboembolism were significantly older than those without procedural thromboembolism (72.3 ± 9.44 vs. 65.7 ± 12.8, *p* = 0.003). AoAC was observed more frequently in the patients with procedural thromboembolism than in those without procedural thromboembolism (22/30 (73.3%) vs. 30/101 (29.7%), *p* < 0.001). Aortic arch type 3 was observed more frequently in the patients with procedural thromboembolism than in those without procedural thromboembolism (11/30 (36.7%) vs. 14/101 (13.9%), *p* = 0.008). The patients with procedural thromboembolism had a shorter time from symptom onset to door than those without procedural thromboembolism (144.1 ± 152.1 vs. 221.6 ± 228.2 min, *p* = 0.034). The procedural time was significantly longer in the patients with procedural thromboembolism than in those without procedural thromboembolism (77.6 ± 37.6 vs. 60.1 ± 29.7, *p* = 0.024).

Multivariable logistic regression analysis for the factors affecting procedural thromboembolism after an MT for an AIS, including age, AoAC, aortic arch type, the time from symptom onset to door, each interventionalist, the timing of the MT, balloon-guided catheter, and procedural time, showed that procedural thromboembolism after an MT for an AIS was independently associated with AoAC (adjusted odds ratios (OR), 6.107; 95% confidence interval (CI), 2.374–15.705; *p* < 0.001) and procedural time (adjusted OR, 1.015; 95% CI, 1.001–1.030; *p* = 0.031) (Table 3).

## 4. Discussion

Complications after an MT for an AIS include a procedural hemorrhagic complication and procedural thromboembolism. When previous studies focused on the efficacy and safety of an MT for an AIS, recanalization was considered a primary outcome, and a procedural hemorrhagic complication was considered a safety outcome [1,15]. However, procedural thromboembolism has not been considered necessary during the MT for an AIS, because the purpose of an MT for an AIS is to achieve the recanalization of the occluded vessels. However, recent advances in MT for an AIS have achieved a recanalization rate of up to 90% [2,3,15], and any complications, including procedural thromboembolism, should not be underestimated and should be studied further. Procedural thromboembolism after an MT for an AIS has rarely been studied, and the study we previously reported is the only study on procedural thromboembolism after an MT for an AIS [1].

In this study, we focused on the relationship between AoAC on a chest X-ray and procedural thromboembolism. During cardiac surgical and interventional procedures, a stroke caused by atherosclerotic plaques of the aortic arch is well known [9,10,11]. However, procedural thromboembolism related to atherosclerotic plaque of the aortic arch during the neurointerventional procedures has rarely been studied. Previous studies on procedural thromboembolism during the neurointerventional procedures have focused on intracranial steps and anti-thrombotic medications, rather than extracranial steps, such as advancing the guiding catheter through the aortic arch [1,4,5,7]. To the best of our knowledge, this is the first study to investigate the relationship between the aortic arch atherosclerosis and procedural thromboembolism after an MT for an AIS.

Our results showed that the overall rate of AoAC was 39.7% (*n* = 52) in patients who underwent an MT for an AIS. As there is no study on the rate of AoAC on a chest X-ray in the general population, it is difficult to compare the rate of AoAC between patients with AIS and the general population. However, considering the poor vascular condition of patients with AIS with tortuous vessels [1], the rate of AoAC on a chest X-ray may be higher in patients with AIS than in the general population. Our results showed the significant relationship between AoAC and aortic arch type, and between procedural thromboembolism and aortic arch type. Poor vascular conditions, such as atherosclerosis, calcification, and tortuous vessels are progressive and change with age [13], which is consistent with our results. The prevalence of atrial fibrillation is also high in old age [16]. These are consistent with our results that the patients with AoAC were older and had more history of atrial fibrillation than those without AoAC.

In this study, procedural thromboembolism after an MT for an AIS was associated with old age, and longer procedural time. Previous studies reported that patients of an older age had poorer vascular conditions than those of a younger age, and the poor vascular condition itself can increase the risk of procedural thromboembolism, and the procedural time can be longer due to poor vascular condition [1]. Additionally, a longer procedural time can also increase the risk of procedural thromboembolism [17], because of a higher incidence of dislodging a thrombus and introducing air bubbles or hydrophilic coating materials during the procedure [12].

This study also showed the strong relationship between AoAC on a chest X-ray and procedural thromboembolism after an MT for an AIS. This can be partially explained by the fact that mechanical stimulation to the aortic arch and adjacent large arteries from the guiding catheter may contribute to artery-to-artery embolization [12], which leads to procedural thromboembolism. Our results suggest that although rapid recanalization is the most important goal of an MT for an AIS, the importance of the careful advancement of the guiding catheter through the aortic arch should not be underestimated to reduce the risk of procedural thromboembolism.

Our results showed that procedural thromboembolism after an MT for an AIS was not significantly associated with each interventionalist and their experiences. Our results also showed that procedural thromboembolism was not significantly associated with timing of the MT. Our results suggest that AoAC on a chest X-ray is the most important procedural thromboembolism after an MT for an AIS, regardless of each interventionalist, the interventionalist’s experiences, and the timing of the MT.

In this study, procedural thromboembolism after an MT for an AIS was not significantly associated with clinical and radiological outcomes. In addition, all the patients with procedural thromboembolism did not show any newly developed neurological symptoms. Therefore, the importance of procedural thromboembolism after an MT for an AIS is controversial. However, as reported in the coil embolization of cerebral aneurysms, although most microembolic infarctions are asymptomatic, they may be associated with brain tissue damage and delayed cognitive dysfunctions [4,5,6,7]. Therefore, the importance of procedural thromboembolism should not be underestimated.

This study defined procedural thromboembolism as new DWI-positive lesions in other territories from the occluded artery on DWI within two days after an MT [1]. It is difficult to distinguish the new DWI-positive lesions in the territory of the occluded artery from the thrombus migration. Therefore, we defined only new DWI-positive lesions in other territories from the occluded artery as procedural thromboembolism. However, new DWI-positive lesions in other territories from the occluded artery can occur not only from procedural thromboembolism but also from new systemic embolic sources. Thus, the definition of procedural thromboembolism after an MT for an AIS remains controversial and it should be discussed more in future studies.

This study has several limitations. First, the study design is retrospective, and it cannot rule out selection bias. However, the selection bias may be low, because all the cases of MT during the study period in our hospital were included. Second, some DWI-positive lesions were difficult to distinguish, and a perfect consensus was not always achieved. Third, the chest X-ray analysis of aortic arch calcification was performed by two neurosurgeons rather than radiologists. The lack of enough experience in analyzing aortic arch calcification on a chest X-ray may be a limitation of this study; however, we tried to make a consensus on the analysis of aortic arch calcification on a chest X-ray. We also tried to confirm the presence of calcification when clear calcification was observed. Forth, our study did not include late thromboembolisms. Our study focused only on the analysis of DWI-positive lesions within two days following an MT for an AIS. More evidence with larger sample sizes and long-term follow-up must confirm these preliminary results.

## 5. Conclusions

In this study, procedural thromboembolism after an MT for an AIS was observed at a relatively high rate; however, none of them showed newly developed neurological symptoms. It was related to AoAC on a chest X-ray and a longer procedural time. This study suggests that more attention is needed in advancing the guiding catheter through the aortic arch during an MT for an AIS in patients with AoAC on a chest X-ray, considering the higher risk of procedural thromboembolism. These preliminary results should be confirmed with further studies with larger sample sizes and long-term follow-up.

## Figures and Tables

**Table 1:** A comparison of patients with and without aortic arch calcification on chest X-ray before mechanical thrombectomy for acute ischemic stroke.

		Aortic Arch Calcification		*p*-Value
		Present (*n* = 52)	Absent (*n* = 79)	
Baseline characteristics	Age in years *	71.3 ± 8.01	64.5 ± 14.0	0.002
	Sex, male (%)	33 (63.5)	46 (58.2)	0.588
	Hypertension (%)	26 (50.0)	36 (45.6)	0.721
	Diabetes mellitus (%)	9 (17.3)	16 (20.3)	0.821
	Atrial fibrillation (%)	24 (46.2)	22 (27.8)	0.040
	Smoking (%)	18 (34.6)	29 (36.7)	0.854
	Aortic arch type			0.001
	Type 1	6	32	
	Type 2	32	36	
	Type 3	14	11	
	Occlusion site			0.739
	Anterior circulation	49	72	
	Posterior circulation	3	7	
	Tandem occlusion (%)	5 (9.6)	13 (16.5)	0.310
	Etiology			0.362
	Cardioembolic	22	27	
	Non-cardioembollic	30	52	
	NIHSS score on admission *	10.9 ± 5.90	11.4 ± 5.85	0.587
	The time from symptom onset to door in min *	176.6 ± 184.7	211.8 ± 232.4	0.241
	The time from door to puncture in min *	151.5 ± 62.8	143.3 ± 50.2	0.405
	IV thrombolysis (%)	28 (53.8)	32 (40.5)	0.154
Procedural details	Each interventionalist			0.444
	Interventionalist 1	36	48	
	Interventionalist 2	13	28	
	Interventionalist 3	3	3	
	Experience of interventionalist (years)	2.65 ± 1.24	2.75 ± 1.21	0.671
	Timing of MT			0.444
	Performed at daytime	38	52	
	Performed at nighttime or on weekends	14	27	
	Approach side, left (%)	27 (51.9)	32 (40.5)	0.375
	Balloon-guided catheter (%)	6 (11.5)	9 (11.4)	1.000
	Intermediate catheter (%)	37 (71.2)	62 (78.5)	0.407
	Rescue treatments (%)	3 (5.8)	6 (7.6)	1.000
	Total number of MT attempts *	2.44 ± 1.78	2.89 ± 2.16	0.220
	Procedural time in min *	69.4 ± 35.3	60.6 ± 30.0	0.125
	Procedural thromboembolism (%)	22 (42.3)	8 (10.1)	<0.001
Clinical and radiological outcomes	Good clinical outcome (%)	18 (60.0)	61 (60.4)	1.000
	Successful recanalization (%)	26 (86.7)	81 (80.2)	0.592

* Data are represented as the mean ± standard deviation. NIHSS: National Institutes of Health Stroke Scale, MT: Mechanical Thrombectomy.

**Table 2:** A comparison of patients with and without procedural thromboembolism after mechanical thrombectomy for acute ischemic stroke.

		Procedural Thromboembolism		*p*-Value
		Present (*n* = 30)	Absent (*n* = 101)	
Baseline characteristics	** Age in years *	72.3 ± 9.44	65.7 ± 12.8	0.003
	Sex, male (%)	16 (53.3)	63 (62.4)	0.401
	Hypertension (%)	14 (46.7)	48 (47.5)	1.000
	Diabetes mellitus (%)	7 (23.3)	18 (17.8)	0.597
	Atrial fibrillation (%)	13 (43.3)	33 (32.7)	0.286
	Smoking (%)	10 (33.3)	37 (36.6)	0.830
	** Aortic arch calcification (%)	22 (73.3)	30 (29.7)	<0.001
	** Aortic arch type			0.018
	Type 1	5	33	
	Type 2	14	54	
	Type 3	11	14	
	Occlusion site			1.000
	Anterior circulation	28	93	
	Posterior circulation	2	8	
	Tandem occlusion (%)	5 (16.7)	13 (12.9)	0.559
	Etiology			0.831
	Cardioembolic	12	37	
	Non-cardioembollic	18	64	
	NIHSS score on admission *	11.9 ± 4.87	11.0 ± 6.12	0.375
	** The time from symptom onset to door in min *	144.1 ± 152.1	221.6 ± 228.2	0.034
	The time from door to puncture in min *	155.8 ± 73.0	143.8 ± 49.2	0.403
	IV thrombolysis (%)	16 (53.3)	44 (43.6)	0.406
Procedural details	Each interventionalist			0.112
	Interventionalist 1	21	63	
	Interventionalist 2	6	35	
	Interventionalist 3	3	3	
	Experience of interventionalist (years)	2.50 ± 1.25	2.77 ± 1.21	0.284
	Timing of MT			0.120
	Performed at daytime	17	73	
	Performed at nighttime or on weekends	13	28	
	Approach side, left (%)	16 (53.3)	43 (42.6)	0.529
	** Balloon-guided catheter (%)	1 (3.3)	14 (13.9)	0.189
	Intermediate catheter (%)	23 (76.7)	76 (75.2)	1.000
	Rescue treatments (%)	2 (6.7)	7 (6.9)	1.000
	Total number of MT attempts *	2.97 ± 2.24	2.63 ± 1.96	0.466
	** Procedural time in min *	77.6 ± 37.6	60.1 ± 29.7	0.024
Clinical and radiological outcomes	Good clinical outcome (%)	18 (60.0)	61 (60.4)	1.000
	Successful recanalization (%)	26 (86.7)	81 (80.2)	0.592

* Data are represented as the mean ± standard deviation. ** Variables for logistic regression model. NIHSS: National Institutes of Health Stroke Scale, MT: Mechanical Thrombectomy.

**Table 3:** Multivariable logistic regression analysis of factors affecting procedural thromboembolism after mechanical thrombectomy for acute ischemic stroke.

Factors	Adjusted OR	Adjusted 95% CI	*p*-Value
Aortic arch calcification	6.107	2.374–15.705	<0.001
Procedural time	1.015	1.001–1.030	0.031

OR: odds ratio; CI: confidence interval.
